# Proper Reporting of Statistical Parameters in Clinical Trials Published in Indian Medical Journals. Is Inclusion of Statistician Play any Significant Role?

**DOI:** 10.4103/0975-1483.80308

**Published:** 2011

**Authors:** Jaykaran, N Chavda, P Yadav

**Affiliations:** *Department of Pharmacology, Govt. Medical College, Surat, India*

Sir,

It is observed that the clinical trials published in various medical journals are poor in reporting of various methodological aspects, including sample size calculation and power.[1] It is difficult to generalize the results of clinical trials having poor reporting of statistics to the normal patient population and conducting these clinical trials raises ethical issues.[2] It is always advisable to take the help of a statistician both during the analysis of data and design of clinical trials.[3] Not associating a statistician while designing clinical trials is associated with the poor reporting of statistical parameters in clinical trials.[3]

This study is designed to check the function of statisticians in clinical trials published in five Indian medical journals and see the impact of employing these statisticians on reporting of some important statistical parameters. Clinical trials published in four Indian medical journals were selected for analysis. Statistical parameters selected for comparisons were the following: calculation of sample size, sample size, calculation of power, appropriate statistical tests, significant *P* value after Bonferroni correction, number of primary endpoints, and calculation of post hoc power of the study.

All clinical trials published in four Indian medical journals in 9 years (January 2000 to December 2008) were downloaded. Each author critically appraised these clinical trials for the various statistical parameters on the basis of a predesigned proforma. Information regarding the role of a statistician was seen at authors’ section, acknowledgement, and methods section of clinical trials. These trials were also appraised for information on calculation of sample size, even partial calculation of sample size were taken into account. Information regarding the exact sample size was noted in the proforma. Authors also surveyed all clinical trials for information regarding calculation of power before starting of study (design phase). Appropriate statistical tests were evaluated based on the aim of the study, type of data, and distribution of data. These clinical trials were also appraised for methods of adjustment of multiple endpoints and whether the *P* value is still significant after adjustment of multiple endpoints on the basis of Bonferroni correction. Endpoints are considered as events or outcomes that can be measured objectively to determine whether the intervention being studied is beneficial. We did not include adverse effects as endpoints of trials. Post hoc power of trials was also calculated at 50% difference between the outcomes with the help of G Power software.[4] Discrepancies observed between the authors were resolved by consensus.

Qualitative data (sample size calculation, power calculation, appropriate statistical tests, significant *P* value after Bonferroni correction and post hoc power calculation (>80%) for the 50% difference in the outcome) were expressed in frequency and percentage. Difference between the group was analyzed by Chi-square test (with Yates correction where appropriate). Quantitative data (sample size, number of primary endpoints) were expressed as mean and difference between the groups analyzed by using unpaired *t* test. Before using unpaired t test, normal distribution of data was confirmed by Skewness, Kurtosis, Kolmogorov Smirnov test, and Shapiro Wilk test. SPSS for Window ver. 17 was used for analysis. Post hoc power was calculated by G Power software.

A total of 68 clinical trials were collected, of which the role of a statistician was acknowledged in 13 trials (19.1%, 95% CI: 11.5%-30%). Of the 13 clinical trials in which the help of a statistician was acknowledged, 8 (61.5%) were positive and in the remaining 55 trials, 27 (49%) were positive. There was statistically significant difference in the number of primary endpoints and sample size between the two groups. Other statistical parameters were not different statistically [Table 1]. Therefore, based on this study, we can conclude that a statistician does not seems to contribute much in correct reporting of statistical parameters, except sample size and number of primary endpoints.

**Table 1:** Difference between various statistical parameters between both groups

Frequencies→ Groups↓	Sample size calculated	Sample size (Mean (SD))	Calculation of Power	Appropriate statistical tests	Significant *P* value after Bonferroni correction	Post hoc >80% power for large difference	Number of Primary endpoints (Mean (SD))
Statistician (n=13)	7 (53.8)	58.8 (24.2)	7 (53.8)	12 (92.3)	9 (69.2)	11 (84.6)	1.42 (0.51)
Non Statistician (n=55)	22 (40)	41.8 (23.3)	20 (36.3)	50 (90.9)	30 (54.5)	41 (74.5)	2.16 (0.89)
*P* value	0.36	0.019	0.24	0.87	0.33	0.48	0.005
Statistical test	Chi-square test	Unpaired t test	Chi-square test	Chi-square test (Yates correction)	Chi-square test (Yates correction)	Chi-square test (Yates correction)	Unpaired t test

Values in parentheses are percentages

This study has some limitations, one of which is its small sample size (Indian medical journals and clinical trials) for analysis. Another limitation here was difficulty in knowing the time of intervention by statisticians, whether he/she has contributed from the start of the clinical trial (design phase) or only during analysis of results. Another limitation may be under-reporting of the role of statistician. In this study, Bonferroni correction was used for adjustment of multiple endpoints. This method of adjustment becomes less reliable, as the number of endpoints increase.
